# Highly efficient cell-type-specific gene inactivation reveals a key function for the *Drosophila* *FUS* homolog *cabeza* in neurons

**DOI:** 10.1038/srep09107

**Published:** 2015-03-16

**Authors:** Marie Frickenhaus, Marina Wagner, Moushami Mallik, Marica Catinozzi, Erik Storkebaum

**Affiliations:** 1 Molecular Neurogenetics Laboratory, Max Planck Institute for Molecular Biomedicine, 48149 Münster, Germany; 2 Faculty of Medicine, University of Münster, 48149 Münster, Germany

## Abstract

To expand the rich genetic toolkit of *Drosophila melanogaster*, we evaluated whether introducing FRT or LoxP sites in endogenous genes could allow for cell-type-specific gene inactivation in both dividing and postmitotic cells by GAL4-driven expression of FLP or Cre recombinase. For proof of principle, conditional alleles were generated for *cabeza* (*caz*), the *Drosophila* homolog of human FUS, a gene implicated in the neurodegenerative disorders amyotrophic lateral sclerosis (ALS) and frontotemporal dementia (FTD). Upon selective expression in neurons or muscle, both FLP and Cre mediated *caz* inactivation in all neurons or muscle cells, respectively. Neuron-selective *caz* inactivation resulted in failure of pharate adult flies to eclose from the pupal case, and adult escapers displayed motor performance defects and reduced life span. Due to Cre-toxicity, FLP/FRT is the preferred system for cell-type-specific gene inactivation, and this strategy outperforms RNAi-mediated knock-down. Furthermore, the GAL80 target system allowed for temporal control over gene inactivation, as induction of FLP expression from the adult stage onwards still inactivated *caz* in >99% of neurons. Remarkably, selective *caz* inactivation in adult neurons did not affect motor performance and life span, indicating that neuronal caz is required during development, but not for maintenance of adult neuronal function.

D*rosophila melanogaster* has been at the forefront of genetic research for over a century now^1^, and a rich toolkit is available for genetic manipulation^2^. This toolkit includes the generation of mutant animals in which a gene of interest is inactivated, either in all body cells, or in homozygous mutant clones^3^,^4^,^5^. Such homozygous mutant clones are ideally suited to study the cell-autonomous effects of loss of gene function, but they can only be generated in dividing cells and typically constitute only a limited fraction of a cell population. The availability of a genome-wide transgenic RNAi library allows for cell-type-specific knock-down of gene expression^6^, but knock-down efficiency is variable and never 100%, and off-target effects may confound the interpretation of observed phenotypes. Recently, a strategy for conditional mutagenesis in *Drosophila* based on CRISPR/Cas9-induced gene disruption in somatic cells was reported^7^. It involves GAL4-driven Cas9 expression in flies that ubiquitously express one or more gRNAs targeting the gene of interest. Although the percentage of GAL4-expressing cells in which gene inactivation occurred was not evaluated, this strategy recapitulated known mutant phenotypes to variable degrees. Apart from the variable phenotypic outcomes and high interindividual variation, a downside of this approach is that the induced mutations are not molecularly defined and distinct mutations may occur in individual target cells, some of which may not represent (full) loss of function alleles.

In an attempt to overcome the limitations of existing methods, we decided to evaluate whether flanking an endogenous gene with either FRT or LoxP recombination sites would allow for efficient cell-type specific gene inactivation in *Drosophila*. We anticipated that excision of a DNA segment bounded by direct FRT repeats may be an efficient way to inactivate *Drosophila* genes, as transgenes in which a promoter is separated from a coding sequence by a stop cassette flanked by FRT sites were previously used in so-called ‘FLP-out' approaches for lineage tracing analysis^8^ or cell-type-specific rescue strategies^9^. Furthermore, a strategy conceptually similar to our approach was previously reported^10^, but only used for analysis of mutant clones and not for inactivation of a gene of interest in all cells of a defined cell population. For prove of concept, we selected the *cabeza* (*caz*) gene, which is the *Drosophila* homolog of human *FUS*. *FUS* is implicated in various human diseases, including the neurodegenerative disorders amyotrophic lateral sclerosis (ALS) and frontotemporal dementia (FTD)^11^. Although FUS shuttles between the nucleus and the cytoplasm, FUS displays a predominantly nuclear localization under physiological conditions. Most familial ALS-associated mutations cluster in the C-terminal nuclear localization signal of FUS, resulting in a shift from nuclear to a more cytoplasmic localization, formation of cytoplasmic FUS-containing protein aggregates, and reduced nuclear FUS levels^12^. It is currently unclear whether ALS-FUS is caused by a “gain-of-toxic-function”, possibly mediated by cytoplasmic aggregates, or by a (partial) loss of nuclear function, or a combination of both mechanisms.

As loss of *FUS* function may contribute to ALS pathogenesis, inactivation of the *Drosophila FUS* homolog *caz* may not only provide insight into the physiological function of *caz*, but may also help unraveling the molecular pathogenesis of ALS-FUS. *Caz* mutant flies were reported previously, with only 14% of caz mutants surviving to adulthood and adult escaper flies exhibiting locomotion defects and shortened life span^13^. Here, we generated two independent *caz* null alleles, as well as conditional *caz* alleles. The conditional *caz* alleles were used to evaluate the efficiency of cell-type-specific gene inactivation in *Drosophila*, as well as the physiological consequences of neuron-selective *caz* inactivation.

## Results

### Generation of *caz* null and conditional alleles

**Figure 1:**
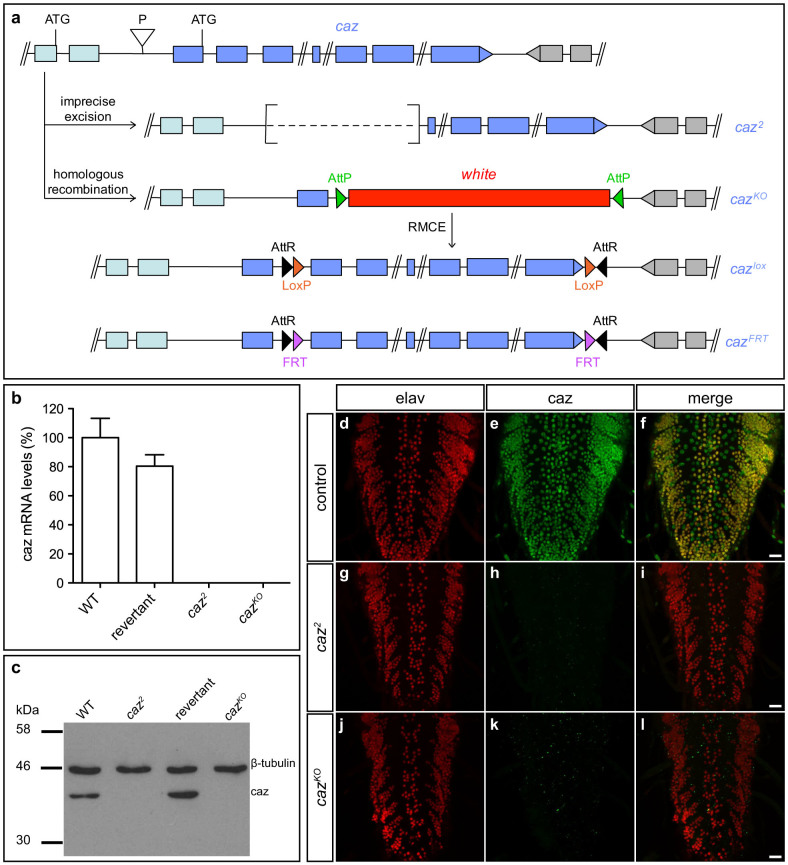
Generation and characterization of *caz* knock-out and conditional knock-out alleles. (a), The *caz*^2^ allele was generated by imprecise excision of the P-transposable element P^Mae-UAS.6.11^ DP00882 in the *caz* gene promoter, which resulted in excision of exons 1 through 3. *Caz^KO^* was generated by homologous recombination, replacing *caz* exons 2 through 7 by a *white* marker gene flanked by AttP sites. ΦC31-mediated RMCE was subsequently used to reintroduce *caz*, flanked by either LoxP (*caz^lox^*) or FRT sites (*caz^FRT^*). (b), Quantitative real-time PCR revealed that *caz*^2^ and *caz^KO^* mutants are *caz* transcript null. The ‘revertant' allele is generated by precise excision of P^Mae-UAS.6.11^ DP00882, and serves as a control for *caz*^2^. N = 5 (c), No caz protein could be detected in *caz*^2^ or *caz^KO^* mutants by Western blot. The full-length blot is shown in Supplementary Figure S5. (d–l), Immunostaining for caz and the neuronal nuclear marker elav revealed caz expression in all neuronal nuclei, as well as in non-neuronal cells in third instar larval ventral cord of control animals. In *caz*^2^ and *caz^KO^* mutants, no caz protein was detected. Scale bar: 20 μm.

To generate *caz* “knock-out” (KO) and conditional KO alleles, the integrase-mediated approach for gene knock-out (IMAGO)^10^ was used (Figure 1a). In the first step, *in vivo* homologous recombination^14^ was used to replace exons 2 through 7 of the *caz* gene by a *white* eye color marker, flanked by *AttP* integrase sites (*caz^KO^* allele). In the second step, ΦC31 integrase-mediated cassette exchange was used to reintroduce the wild type *caz* genomic sequences, either without or with flanking LoxP or FRT sites (*caz^AttR^, caz^lox^ and caz^FRT^* alleles; Figure 1a). An independent *caz* null allele was generated by imprecise excision of a P-transposable element in the *caz* gene promoter. This yielded the *caz*^2^ allele, in which exons 1 through 3 of *caz* are deleted (Figure 1a). Quantitative real-time PCR (qPCR), Western blotting and immunostaining revealed that both the *caz^KO^* and the *caz*^2^ alleles are transcript and protein null (Figure 1b–l). Immunostaining further revealed that in third instar larval central nervous system (CNS), caz is expressed panneuronally, as well as in non-neuronal cells (Figure 1d–f).

### Loss of *caz* function results in motor defects

**Figure 2:**
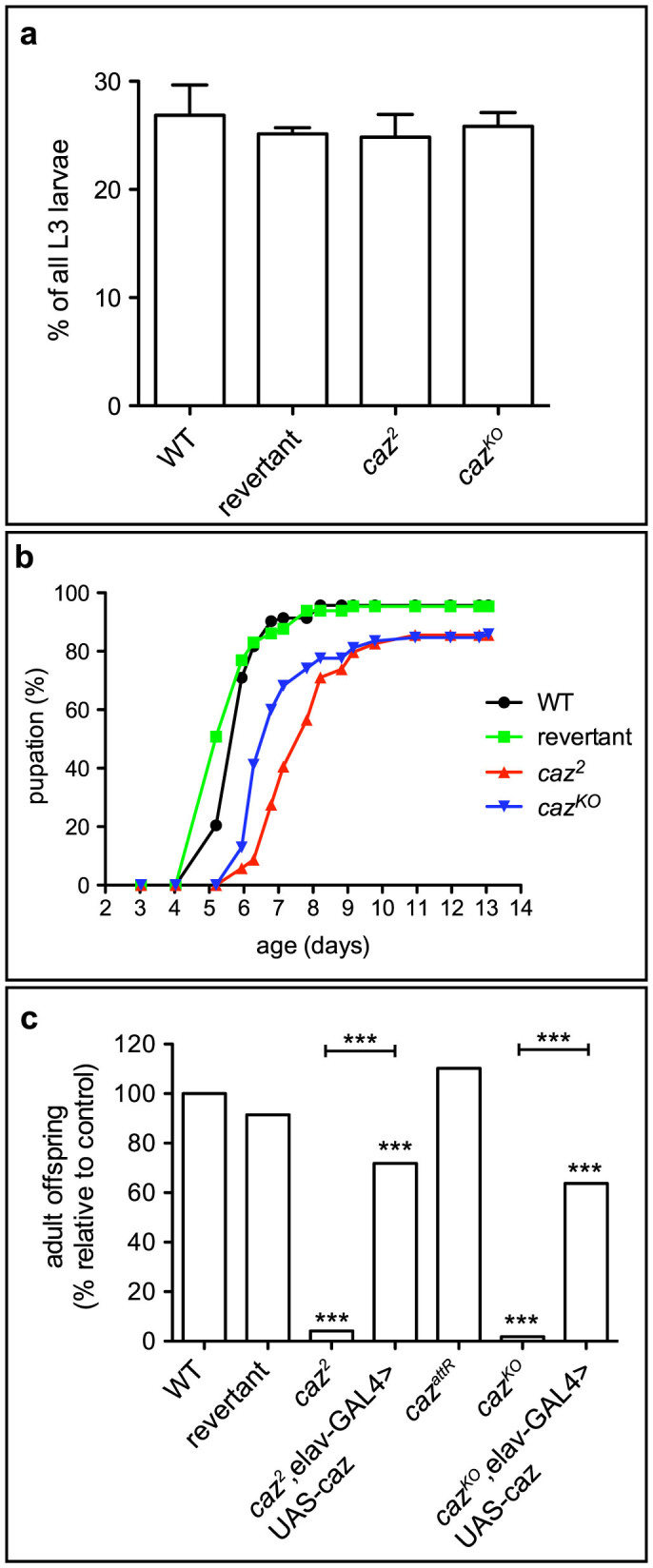
*Caz*^2^ and *caz^KO^* mutants display developmental delay and pupal lethality, which can be rescued by neuron-selective caz reintroduction. (a), At the third instar larval stage, *caz*^2^ and *caz^KO^* mutant animals are present at the expected Mendelian ratios. Results of three independent experiments were pooled, with 300–400 L3 larvae per experiment. (b), *caz* mutants display an increased time to pupation, indicative of developmental delay. N = 80–110. (c), *caz*^2^ and *caz^KO^* mutant animals died during the pupal stage, with very few adult escapers. Pupal lethality could be rescued by selective expression of wild type *caz* in neurons (elav-GAL4). N = 92–393.

As *caz* is on the X chromosome, hemizygous males were used to study *caz* null phenotypes. Loss of *caz* function resulted in developmental lethality, as no *caz* mutant adult flies emerged. At the third instar larval stage, *caz* mutant larvae were present at the expected Mendelian ratio (Figure 2a), but *caz* mutant animals took significantly more time than control animals to reach the pupal stage, indicative of a developmental delay (Figure 2b). Thus, loss of *caz* function results in developmental lethality during the pupal stage, with at least a fraction of animals developing to pharate adults. The latter fail to eclose from the pupal case, with only very few adult escapers (Figure 2c, Movie S1 and S2). When partially eclosed pharate adults were dissected out of their pupal case, these flies displayed severe motor coordination phenotypes (Movie S3) and died within a day. *Caz^AttR^* flies, in which the *caz^KO^* allele was restored to wild type, did not show any pupal lethality or adult eclosion defects (Figure 2c), demonstrating that *caz^KO^* phenotypes are due to specific inactivation of the *caz* gene. Similar to previously reported results^13^, *caz* null phenotypes could be rescued by selective reintroduction of wild type caz transgenic protein in neurons by elav-GAL4 (Figure 2c), demonstrating that loss of *caz* function in neurons is necessary to induce pupal lethality and adult eclosion defects.

### Neuron-selective *caz* inactivation impairs motor performance and reduces life span

**Figure 3:**
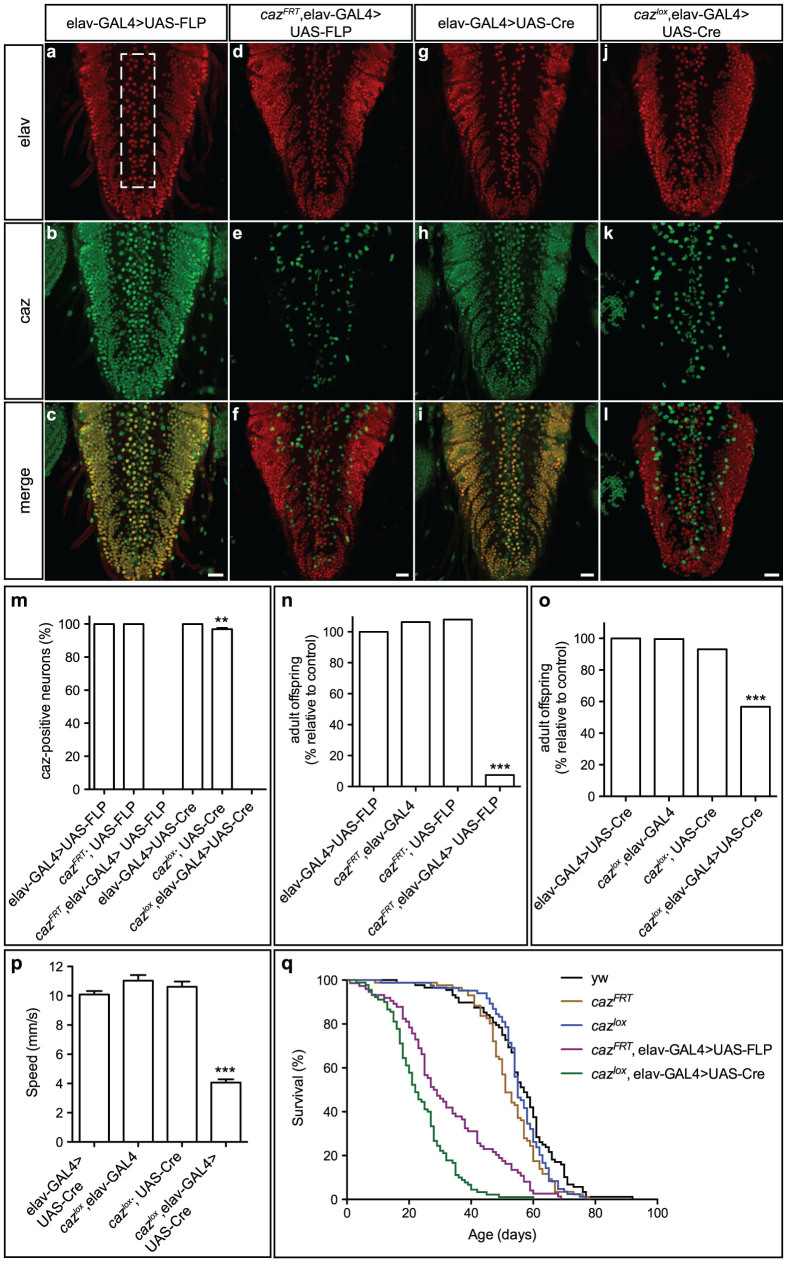
Neuron-selective *caz* inactivation induces pupal lethality, motor deficits and reduced life span. (a–l), elav-GAL4 was used to drive panneuronal expression of FLP (a–f) or Cre (g–l), either in a wild type or conditional *caz* KO background. Immunostaining for caz and elav on third instar larval ventral nerve cord revealed *caz* inactivation in all neurons, but not in non-neuronal cells. Scale bar: 20 μm. (m), Quantification confirmed *caz* inactivation in 100% of neurons. N = 7–13. (n, o), Adult offspring frequencies of control genotypes versus neuron-selective *caz* KO mediated by FLP/FRT (n) or Cre/LoxP (o) revealed developmental lethality with a variable fraction of adult escapers. N = 514–665 (n) and 414–540 (o). (p), Neuronal *caz* KO adult escaper flies display significant motor performance deficits, as revealed by a negative geotaxis climbing assay. N = 100. (q), Neuronal *caz* KO adult escaper flies display reduced life span. N = 74–90.

To evaluate whether conditional gene inactivation in all or a large proportion of neurons is possible in *Drosophila*, the elav-GAL4 driver was used to express either FLP or Cre recombinase panneuronally in *caz^FRT^* or *caz^lox^* animals, respectively. Remarkably, FLP/FRT-mediated recombination resulted in loss of caz protein in all neurons in the third instar larval CNS (Figure 3a–f). This was confirmed by quantitative analysis of *caz* inactivation in an anatomically defined population of neurons in the ventral nerve cord (Figure 3m). Similarly, Cre/LoxP-mediated recombination also resulted in loss of caz protein in all CNS neurons (Figure 3g–m). Of note, consistent with previous reports^15^, panneuronal Cre expression induced a mild rough eye phenotype (Supplementary Figure S1).

Selective inactivation of *caz* in all neurons resulted in pupal lethality attributable to the inability of adult flies to eclose from the pupal case (Movie S4, S5), with a variable fraction of adult escapers (Figure 3n, o). FLP/FRT-mediated neuronal *caz* inactivation resulted in only 7.5% of adult escapers, whereas Cre/LoxP-mediated recombination resulted in 56.7% of the expected frequency of adult offspring eclosing. We speculate that this discrepancy may be due to differences in timing of *caz* inactivation and/or genetic background. Nonetheless, independent of the recombinase system used, neuronal *caz* inactivation leads to motor defects, and small differences in the degree of motor impairment may result in an apparently substantial difference in adult escaper frequencies. Overall, to a large extent, the neuronal *caz* KO phenocopies the full body KO, although the neuronal *caz* KO phenotype seemed somewhat less severe, suggesting that loss of *caz* function has deleterious effects in cell types other than neurons, which contribute to the *caz* mutant phenotypes. Evaluation of *caz* neuronal KO adult escaper flies in a negative geotaxis climbing assay revealed significant motor deficits (Figure 3p, Movie S6), and the life span of these flies was significantly reduced (Figure 3q, Supplementary Figure S2).

**Figure 4:**
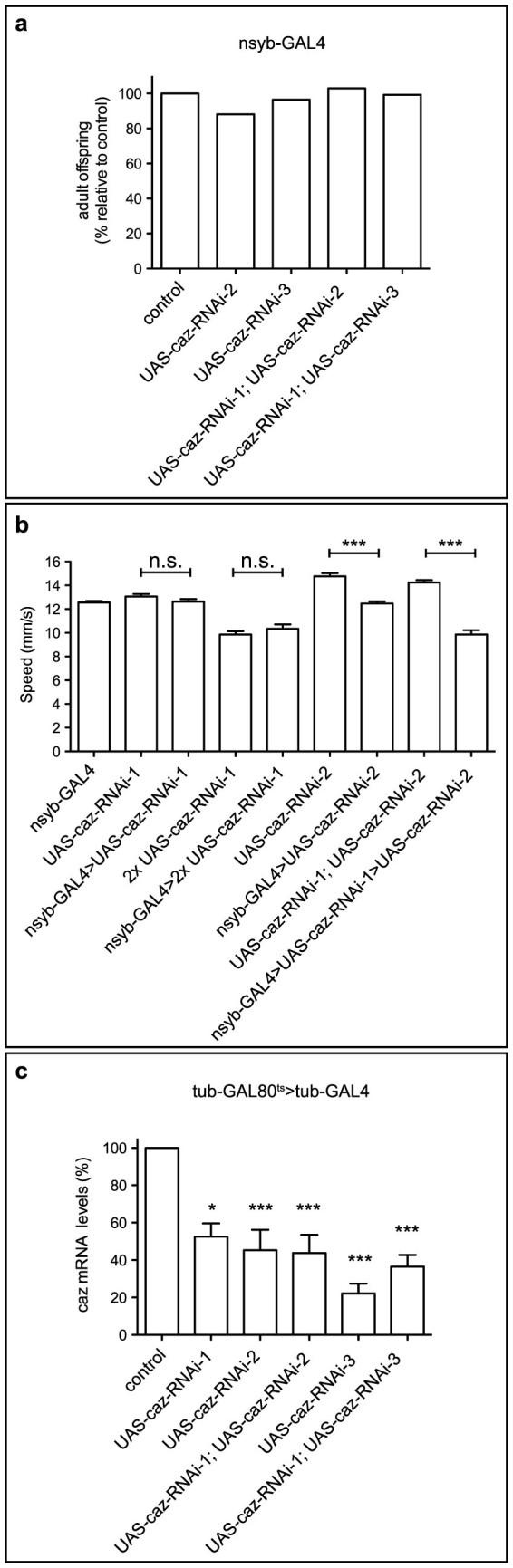
Neuronal caz knock-down by transgenic RNAi does not induce developmental lethality and does not or only slightly impair motor performance. (a), Panneuronal expression of caz-RNAi with nsyb-GAL4 does not induce significant developmental lethality. Data normalized to control (w1118) are shown. N = 83–144. (b), Panneuronal caz knock-down by nsyb-GAL4 induced no or only slight motor performance defects in a negative geotaxis climbing assay. (c), caz transcript levels were quantified by qPCR in heads of adult flies ubiquitously expressing caz-RNAi from the adult stage onwards. Three distinct transgenic caz-RNAi lines were tested, either as a single transgene or in combinations of two transgenes. Significant caz knock-down of ≈ 50 to 80% was observed. caz-RNAi-1: VDRC100291; caz-RNAi-2: HMS00790; caz-RNAi-3: HMS00156.

In contrast to panneuronal *caz* inactivation, panneuronal *caz* knock-down by two independent transgenic RNAi lines did not induce developmental lethality and did not or only slightly impair motor performance (Figure 4a, b). This can be explained by incomplete *caz* knock-down (Figure 4c), illustrating the advantage of cell-type specific gene inactivation over transgenic RNAi knock-down.

### Selective inactivation of *caz* in muscles

**Figure 5:**
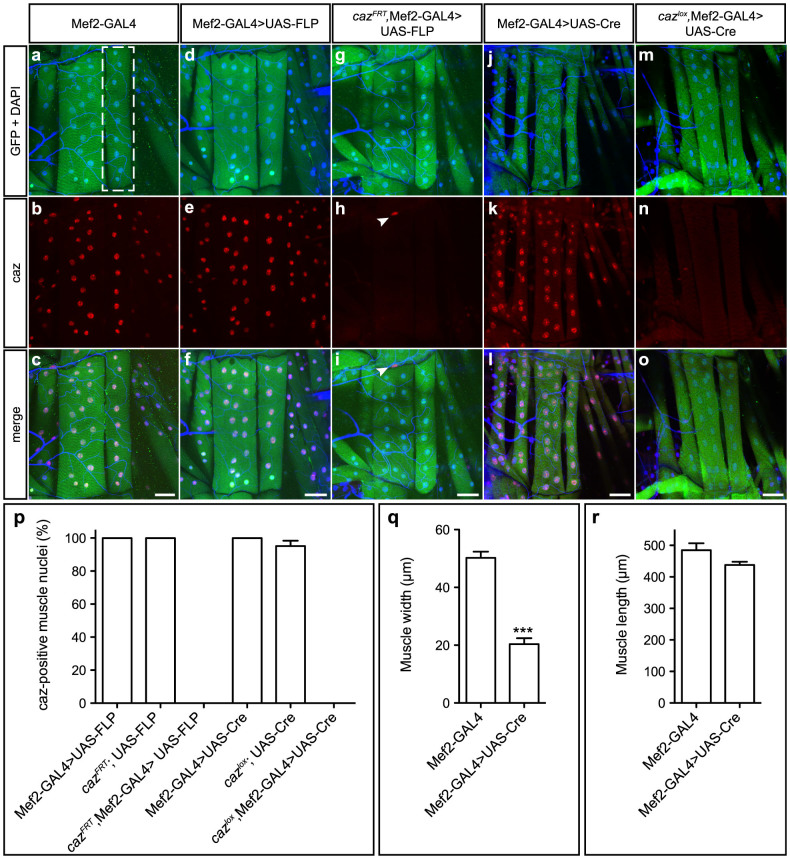
Muscle-selective *caz* inactivation. (a–o), Mef2-GAL4 was used for muscle-selective expression of FLP or Cre, either in wild type or conditional *caz* KO background. GFP co-expression was used to visualize muscle cells and DAPI to label nuclei. Immunostaining for caz and GFP on third instar larval body wall musculature revealed *caz* inactivation in all muscle cells. Arrowheads in panels h and i indicate caz expression in a non-muscle cell. Scale bar: 50 μm. (p), Quantification confirmed *caz* inactivation in 100% of muscle cells. Muscle 7 (indicated in panel a) in segments A1 and A2 was used for quantification. N = 7–10. (q, r), Defects in muscle morphology induced by Cre expression are documented by reduced width of muscle 7 (q), whereas muscle length was not significantly altered (r). N = 8–9.

To evaluate whether conditional gene inactivation also works in cell types other than neurons, selective gene inactivation in muscle cells was tested. We expected selective gene inactivation in muscle to be particularly challenging, as mature muscle fibers in *Drosophila* develop through fusion of myoblasts^16^. Therefore, target gene excision should occur in all nuclei, in order to have no residual gene expression in mature myocytes. Mef2-GAL4 was used to drive expression of either FLP or Cre in myoblasts of *caz^FRT^* or *caz^lox^* animals, respectively. Remarkably, in third instar larval body wall muscles, this approach resulted in loss of caz protein in all muscle cells (Figure 5a–p). Whereas FLP expression in muscles did not induce any obvious morphological changes, Cre expression resulted in a significantly reduced muscle width (Figure 5q, r), indicating that Cre expression is toxic to muscle cells.

### The GAL80 TARGET system allows for temporal control over cell-type-specific gene inactivation

**Figure 6:**
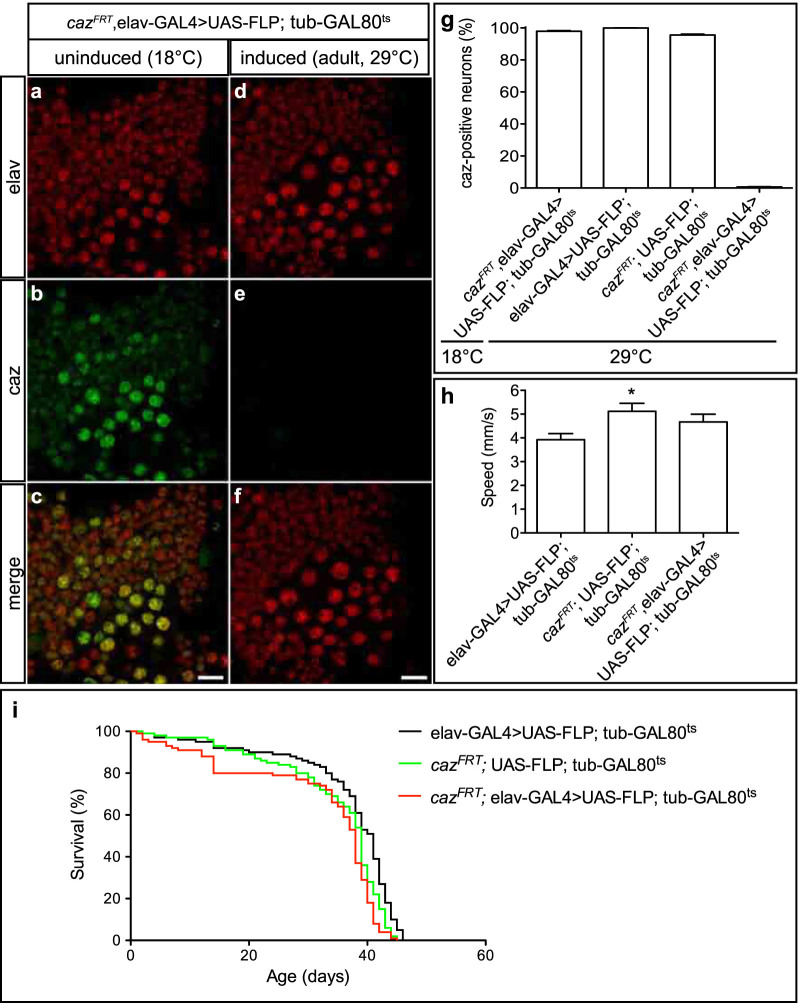
Temporal control over neuron-selective *caz* inactivation. Tubulin-GAL80^ts^>elav-GAL4 was used for induction of FLP expression in neurons of adult *caz^FRT^* flies. (a-f), Immunostaining for caz and elav on adult ventral cord revealed highly efficient *caz* inactivation in adult neurons (scale bar: 10 μm). (g), Quantification documented *caz* inactivation in >99% of adult neurons. N = 9–11. (h), Loss of neuronal *caz* function from the adult stage onwards does not affect motor performance at 4 weeks of age, as revealed by a negative geotaxis climbing assay. N = 90–100. (i), Loss of caz in adult neurons does not reduce life span in a biologically relevant manner. N = 100.

Finally, to determine if we could gain temporal control over cell-type-specific gene inactivation, the GAL80 TARGET system^17^ was used to repress elav-GAL4-driven FLP expression during development, and induce panneuronal FLP expression from the adult stage onwards. Two weeks after induction, *caz* was inactivated in more that 99% of neurons (average ± SEM: 99.38 ± 0.22%; Figure 6a–g). Thus, selective gene inactivation in neurons is feasible in *Drosophila* adults. This approach is particularly relevant for genes for which panneuronal inactivation during development results in lethality or developmental defects, precluding proper analysis of gene function in adult neurons.

As ALS is an adult-onset disease, we reasoned that it would be particularly relevant to evaluate the effect of neuronal loss of *caz* function from the adult stage onwards on motor performance and life span. Surprisingly, loss of caz in adult neurons did not result in a climbing defect at four weeks of age (Figure 6h), and the life span of these flies was comparable to controls (Figure 6i). These data indicate that neuronal *caz* function is not required for maintenance of adult neuronal function.

## Discussion

The approach presented here allows for highly efficient inactivation of endogenous *Drosophila* genes in defined cell populations and analysis of resulting phenotypes. This method can be used in both dividing and non-dividing cells to address a broad range of biological questions, and it allows for temporal control over gene inactivation. Consistent with previously described Cre toxicity in mice^18^,^19^,^20^,^21^,^22^,^23^ and *Drosophila*^15^, panneuronal Cre expression induced a mild rough eye phenotype and muscle expression resulted in severe muscle phenotypes. Moreover, the UAS-Cre transgenes used in this study displayed leaky expression (Supplementary Figure S3). Therefore, FLP/FRT is the preferred system for cell-type-specific gene inactivation in *Drosophila*. We believe that selective gene inactivation in a specific cell type complements experiments in which a mutant phenotype is rescued by cell-type-specific reintroduction of wild type protein. Indeed, the latter approach allows for evaluating whether loss of gene function in a certain cell type is necessary to induce (aspects of) the mutant phenotype, whereas cell-type-specific gene inactivation evaluates whether loss of gene function in a certain cell type is sufficient to induce phenotypes. There are two caveats when using cell-type specific rescue approaches. Firstly, the UAS-transgene may be leaky, what may confound the interpretation of results. This also seems to be the case for the UAS-caz transgene used in this study, as the mere presence of the transgene results in a slight rescue of pupal lethality (Supplementary Figure S4). Secondly, the levels of the rescue-transgene may not match the physiological protein levels in the cell type of interest. In the scenario where physiological protein levels would rescue the mutant phenotype, too low transgene expression levels may fail to do so, whereas transgene expression higher than physiological levels may induce overexpression phenotypes, which may again preclude rescue of mutant phenotypes.

Compared to cell-type-specific knock-down by transgenic RNAi, cell-type-specific gene inactivation has the advantage that it results in complete loss of gene function in the targeted cells, and that there is no risk for off-target effects. This is illustrated by the finding that panneuronal *caz* inactivation induced much stronger phenotypes than panneuronal caz knock-down (Figure 4). Compared to a recently reported strategy for conditional mutagenesis in *Drosophila* based on CRISPR/Cas9-induced gene disruption in somatic cells^7^, our approach has the advantage that the cell-type-specific genetic mutations are molecularly defined, identical in all target cells and always resulting in complete loss of gene function. As a consequence, resulting phenotypes are less variable and more consistent between different individuals.

For introduction of FRT or LoxP sites in the *caz* gene, we have used the IMAGO approach^10^, which is labor-intensive and time-consuming. Recently however, several synthetic nuclease-based methods have been reported for genome engineering in *Drosophila*, including zinc finger nucleases, TALENs and CRISPR/Cas9^24^,^25^. These nuclease-based systems are highly efficient and allow for fast and easy genomic engineering. In particular, a number of recent publications report that CRISPR/Cas9 technology allows for introduction of precise genomic changes within a time frame of one month^26^,^27^,^28^,^29^. Thus, this approach should allow for facile generation of conditional alleles. Therefore, cell-type specific gene inactivation in *Drosophila* can be expected to become a routine genetic manipulation strategy in the near future.

Finally, as *caz* is the *Drosophila* homolog of human FUS, our findings may be relevant for ALS pathogenesis. Although ALS-FUS is characterized by a dominant inheritance pattern, postmortem neuropathological analysis revealed cytoplasmic FUS-immunoreactive inclusions in neurons and glial cells, often accompanied by loss of FUS from its normal nuclear localization^30^,^31^,^32^. Furthermore, cell-biological studies have shown that most of the ALS mutations induce a shift in FUS subcellular localization from the nucleus to the cytoplasm, and a positive correlation exists between the degree to which FUS nuclear import is impaired and the age of disease onset and the rate of disease progression^33^,^34^,^35^,^36^,^37^. Loss of nuclear FUS function may therefore be implicated in ALS-FUS pathogenesis. Both *caz* mutant lines described in this study die during the pupal stage of development, with at least a fraction of pharate adults trying to eclose from the pupal case, but failing to do so due to motor incapability. The *caz* mutant pupal eclosion defect could be rescued by selective reintroduction of wild type caz transgenic protein in neurons, demonstrating that loss of caz function in neurons is necessary to induce adult eclosion defects. Overall, these findings are in line with the phenotypes of a previously described *caz* mutant, which were interpreted as supporting the hypothesis that loss of FUS function may causally contribute to ALS-FUS pathogenesis^13^.

We could further demonstrate that selective *caz* inactivation in all neurons during development (elav-GAL4^C155^ drives transgene expression from embryonic stage 12 onwards^38^) results in adult motor deficits and reduced life span. In contrast, selective neuronal *caz* inactivation from the adult stage onwards did not result in motor performance defects at four weeks of age, and life span was not affected. The latter result may be particularly relevant in the context of ALS, which is an adult-onset disease. Together, our findings indicate that neuronal caz is not required for maintenance of adult neuronal function. Rather, loss of neuronal caz function during development is necessary and sufficient to induce adult motor performance defects and shortened life span. Further studies are needed to evaluate whether loss of neuronal caz during development leads to neurodevelopmental defects or rather to degenerative changes during development.

Although we are careful with extrapolating findings from Drosophila models to human ALS patients, our data suggest that (partial) loss of neuronal FUS function may not be sufficient to cause adult motor neuron degeneration in ALS-FUS patients. Rather, a gain of toxic function by cytoplasmic FUS mislocalization and/or aggregation may be necessary to cause motor neuron degeneration. An alternative possibility is that loss of FUS function in non-neuronal cells in addition to neuronal loss of FUS function is necessary to cause disease. Non-cell-autonomous contributions to motor neuron degeneration have been clearly demonstrated in mutant SOD1 mouse models for ALS^39^. In any case, our data do not exclude the possibility that loss of nuclear FUS function could causally contribute to motor neuron degeneration in ALS-FUS patients. Loss of neuronal FUS function may even be a necessary contributor to ALS pathogenesis, albeit not sufficient to cause motor neuron degeneration by itself.

## Methods

### Molecular cloning

All PCR primer sequences are listed in Supplementary Table S1. To introduce homology arms into pP{white-STAR}^10^ for homologous recombination, 4590 bp of sequence upstream of base 21 in intron 1 of *caz* were PCR-amplified (Phusion polymerase, Thermo Scientific) and cloned into pP{white-STAR} using PCR primers 5′HR_FW and 5′HR_REV followed by restriction digestion of the PCR product with KpnI and XbaI. pP{white-STAR} was digested with KpnI and AvrII (compatible ends with XbaI) and ligated to the digested PCR product. To introduce the 3′ homology arm, 4990 bp of sequence, beginning 10 bp downstream of the *caz* gene, were amplified using 3′HR_FW and 3′HR_REV followed by digestion with NheI and AbsI. pP{white-STAR} containing the 5′ homology arm was then digested with SpeI and XhoI and ligated to the digested 3′HR PCR product (compatible ends).

To facilitate cloning of cassette exchange constructs into pABC, an XbaI restriction site was introduced into pABC-ato^10^ by site-directed mutagenesis using primers pABC_XbaI_FW and pABC_XbaI_REV (mutagenesis PCR with Pfu ultra, Stratagene). To reintroduce wild type caz (*caz^attR^* stock), the caz *gene* was amplified by PCR using primers XbaI_caz_FW and KpnI_caz_REV with P[acman] BAC CH322-28D08 (BACPAC Resources) as a template (Phusion polymerase, Thermo Scientific) and cloned into pABC-ato-XbaI with XbaI and KpnI, replacing ato by caz.

To flank *caz* by FRT and loxP sites (*caz^lox^* and *caz^FRT^* stocks), the recombination sites were first cloned into pABC using synthetic oligonucleotides containing FRT or loxP sites and restriction sites (2xFRT FW and REV, 2xloxP FW and REV, Supplementary Table S1). BsiWI and HindIII were used to digest pABC-ato and the annealed oligonucleotides followed by a ligation. XbaI and KpnI were then used to clone *caz* between the loxP sites (caz PCR product using XbaI_caz_FW and KpnI_caz_REV). NcoI and KpnI were used to clone *caz* between FRT sites (caz PCR product using NcoI_caz_FW and KpnI_caz_REV).

All inserts were fully sequenced before plasmid injections into embryos.

### Generation of fly lines

To generate *caz* null flies by imprecise P-element excision, Ki^1^ p^p^ P[ry Δ2-3]99B transposase (Bloomington stock 4368) was used to mobilize P{y[+ t7.7] = MaeUAS.6.11}DP00882 inserted upstream of the caz gene (Bloomington stock 21828), according to the protocol published by Hummel and Klämbt^40^. Excision events were identified using the *yellow* marker. Candidate excisions were balanced over FM7 and stocks that were not homozygous viable were screened for rescue of lethality with a genomic *caz* construct^13^ and further characterized by PCR genotyping and sequencing. PCR and sequencing with primers caz -1500 FW and caz intron3 REV were used to molecularly characterize the *caz*^2^ imprecise excision allele, revealing that nucleotides 16288762 to 16289716 were deleted*.*

For homologous recombination, the ends-out targeting vector pP{white-STAR} containing a *white* gene flanked by homology arms was introduced into the fly genome using standard transposase-mediated random integration followed by selection of transgenic flies based on red eye color. All plasmid injections were performed by the Model Systems Genomics service at Duke University. The ends-out homologous recombination procedure was based on the protocol of Maggert et al.^41^.

Virgins from stocks carrying the donor construct on chromosome 2 were crossed to males carrying heat-inducible FLP and SceI (P{ry[+ t7.2] = 70FLP}23 P{v[+ t1.8] = 70I-SceI}4A/TM6, Bloomington stock 6935). Parents were transferred every 2-3 days and larvae were heat-shocked in a water bath at 38°C for 1 h one day after the parents had been transferred. All balancer-negative virgins from these crosses were collected and testcrossed to males expressing FLP (P{ry[+ t7.2] = 70FLP}10, Bloomington stock 6938; in total approximately 1300 crosses with 1–2 virgins each were set up). Red-eyed virgins from these crosses were used to establish candidate stocks. Correct targeting events were identified by (i) identification of X-chromosomal white insertions by crossing to FM7/Y males, (ii) screening for lethality of FM7-negative males in candidate stocks (known phenotype of *caz* mutants^13^), (iii) rescue of observed lethality using a genomic *caz* construct^13^, (iv) PCR genotyping of *caz* and surrounding genomic regions using LongAmp Taq polymerase (NEB) and sequencing and (v) Southern Blotting.

To replace the *white* marker gene in *caz^KO^* flies by recombinase-mediated cassette exchange, *caz^KO^* embryos expressing ΦC31 integrase in the germ line (M(vas-int.B)ZH-102D, Bloomington stock 23649) were injected with pABC-caz constructs. Selection of flies with a correct cassette exchange was based on loss of red eye color, followed by PCR genotyping using primers caz RMCE FW, caz RMCE REV1 and caz RMCE REV2 (Supplementary Table S1), which also revealed the direction of the inserted *caz* sequence.

### Cell-type-specific *caz* inactivation

To induce cell-type-specific *caz* knock-out, *caz^lox^* and *caz^FRT^* were recombined with elav-Gal4 (P{w[+ mW.hs] = GawB}elav[C155], Bloomington stock 458) or Mef2-Gal4 (kindly provided by Frank Schnorrer). Homozygous recombinant females were subsequently crossed to UAS-Cre (UASt-Cre II.3 provided by Christian Lehner^15^) or UAS-FLP (P{w[+ mC] = UAS-FLP1.D}JD1, Bloomington stock 4539) males and male offspring was used for experiments. P{w[+ mC] = UAS-GFP.nls}8 was used to express GFP in muscles (Bloomington stock 4776).

To inactivate *caz* panneuronally from the adult stage onwards, tubulin-GAL80^ts^ (Bloomington stock 7018) was used to express UAS-FLP in a temperature-dependent manner. *caz^FRT^*,elav-GAL4; UAS-FLP; tubulin-GAL80^ts^ flies and controls were raised at 18°C to suppress UAS-FLP expression and shifted to 29°C 2 to 4 days after eclosion to excise *caz* in adult neurons. Uninduced controls were not shifted to 29°C but kept at 18°C. Two weeks later, all genotypes were kept at 18° for 6 more days prior to dissection.

### Immunostaining and microscopy

Third instar larval CNS was fixed in 2% PFA for 30 min, followed by three 15 min washes in PBT (1% TritonX-100 in PBS) at RT and blocking with 10% goat serum in PBT for 1 h. Anti-elav (DSHB, 9F8A9, 1:200) and anti-caz (mouse monoclonal clone 3F4, hybridoma line provided by David Ron^42^, 1:30) were diluted in 10% goat serum in PBT and applied overnight at 4°C. After three 20 min washes in PBT, secondary goat anti-mouse antibodies (Alexa Fluor 568 and 488, 1/500) were applied for 2 h at RT, followed by mounting on a microscopy slide in Vectashield medium. Quantification of efficiency of *caz* excision in L3 CNS was performed on 70 neurons organized in central clusters in the ventral nerve cord (Figure 3a). Adult CNS was fixed in 2% PFA for 1 h followed by an immunostaining according to the protocol described for L3 CNS. To quantify the efficiency of *caz* excision in adults, an area of 80 × 80 μm located laterally in between segment T1 and T2 in the ventral nerve cord was used (in a randomly selected layer of a Z stack).

To image larval body wall muscles, third instar larval filets were fixed in Bouin's solution for 3 min followed by three 10 min washes in PBT (0.2% TritonX-100 in PBS) at RT and blocking with 10% goat serum in PBT for 1 h. Anti-GFP (Invitrogen, A6455, 1/1000) and anti-caz (3F4, 1:30) were applied overnight at 4°C in 10% goat serum in PBT. After incubation with secondary goat anti-mouse antibodies (Alexa Fluor 568 and 488, 1/500) for 2h and washing, filets were mounted in Vectashield medium with DAPI to stain muscle nuclei. Quantification of efficiency of *caz* excision in muscles was performed on nuclei of muscle 7 on both sides in segments A1 and A2 (Figure 5a). All images were acquired using a Zeiss LSM700 confocal microscope.

### Western blotting

SDS-PAGE (10% gel) was used to separate third instar larval CNS protein extracts, followed by semi-dry transfer to a PVDF membrane following standard procedures. Primary antibodies against caz (3F4, 1:100) and β-tubulin (E7, DSHB, 1:700) were used, followed by secondary HRP-conjugated anti-mouse IgG (W402B, Promega, 1:2500).

### Drosophila adult offspring frequencies, life span and motor performance assays

Flies for offspring quantifications, life span and motor performance assays were kept at 25°C in a 12h light/dark rhythm on standard *Drosophila* medium. To determine adult offspring frequencies, the F1 generation eclosing from crosses was counted for 9 days starting from the first day of eclosion. The ratio of FM7 negative males to FM7-positive males ( = *caz* mutant, *caz* conditional knock-out or control males versus balancer control males) was calculated and normalized to the ratio of one control genotype. Adult offspring frequencies are reported as percentage relative to the control genotype (set at 100%). The UAS-caz line used for rescue experiments was the UAS-flag-caz line described by Wang et al^13^.

For life span assays, 74–100 males per genotype were collected within 24 h of eclosion and grouped into batches of 10 flies per food vial. The number of dead flies was counted every day and flies were transferred to fresh food vials every 3 days.

For motor performance assays, male flies were collected within 48 h after eclosion and divided into groups of 10 individuals. Motor performance of 6- to 7-day old flies was evaluated. On the day of the motor performance assay, flies were transferred into the test tubes without anesthesia and assayed within 10 minutes under standardized light conditions. 10 test tubes were loaded into a rack, which was mounted onto an apparatus that releases the rack from a fixed height upon pushing a button. The rack falls down, thereby shaking the flies to the bottom of the test tubes and inducing a negative geotaxis climbing response. The whole procedure was videotaped with a Nikon D3100 DSLR camera and repeated 2 more times. The resulting movies were then converted into 8-bit grayscale TIF image sequences with 10 frames per second. Subsequently, the image sequence was imported in ImageJ/FIJI, background-subtracted, filtered and binarized to allow for tracking of flies using the MTrack3 plug-in. Average climbing speed (mm/s) was determined and compared between genotypes.

### Measurement of transcript levels

The efficiency of *caz* knock-down by transgenic RNAi was examined by quantitative real-time PCR (qPCR). Flies expressing caz-RNAi transgenes (Bloomington stock numbers 34839 and 32990 and VDRC stock 100291) under control of the *tub-Gal80^ts^*; *tub-Gal4* target system were raised at 18°C to suppress transgene expression and shifted to 29°C for 48 h to induce transgene expression. Total RNA was extracted from 20 heads per genotype by using the NucleoSpin® RNA kit (Macherey-Nagel). First-strand synthesis was performed on 1 microgram total RNA using the QuantiTect Reverse Transcription Kit (Qiagen). qPCR primer and probe sets designed to amplify caz cDNA were obtained from Applied Biosystems. Measurements were normalized to *EfTuM* and *Actin5C* controls and data were analyzed using the comparative C_T_ method.

### Statistics

Offspring frequency results were analyzed by Chi Square test in Microsoft Excel. Results from climbing assays were analyzed by Mann Whitney U test comparing climbing speeds of individual flies per genotype and per run. Subsequently, Fisher's combined probability test was used to combine the three p values originating from three consecutive measurements per genotype. qPCR results were analyzed by ANOVA followed by Bonferroni correction. For life span analysis, Log Rank Test was used to test for statistical significance. Muscle size results were analyzed by Mann Whitney U test. If not stated otherwise, GraphPad Prism 5 was used to perform statistics and to generate graphs. Bar graphs with error bars represent averages ± SEM.

## Supplementary Material

Supplementary Information Supplementary information

Supplementary Information Movie S1

Supplementary Information Movie S2

Supplementary Information Movie S3

Supplementary Information Movie S4

Supplementary Information Movie S5

Supplementary Information Movie S6
