# Supplementary material for: Highly efficient cell-type-specific gene inactivation reveals a key function for the Drosophila FUS homolog cabeza in neurons
Source: Sci Rep. 2015 Mar 16;5:9107. doi: 10.1038/srep09107 (PMC5390904; doi:10.1038/srep09107)
Supplement: Supplementary Information [file srep09107-s1.pdf]

HIGHLY EFFICIENT CELL-TYPE-SPECIFIC GENE INACTIVATION REVEALS A KEY FUNCTION FOR THE *DROSOPHILA FUS* HOMOLOG *CABEZA* IN NEURONS

Marie Frickenhaus, Marina Wagner, Moushami Mallik, Marica Catinozzi and Erik Storkebaum

SUPPLEMENTARY INFORMATION

SUPPLEMENTARY FIGURE LEGENDS

SUPPLEMENTARY FIGURE S1:

Rough eye phenotypes in elav-GAL4>UAS-Cre flies. Five-day old female flies kept at 25°C are shown.

SUPPLEMENTARY FIGURE S2:

Additional control genotypes for life span of neuronal caz KO adult escaper flies. **a,** The mere presence of FRT or LoxP sites in the *caz* gene, or the additional presence of elav-GAL4, UAS-FLP or UAS-Cre does not influence life span in a biologically relevant manner. ***b,*** Panneuronal FLP or Cre expression does not reduce life span. Following genotypes are also shown in Figure 2q: yw, caz^{FRT} and caz^{lox}.

SUPPLEMENTARY FIGURE S3:

Leaky Cre expression results in occasional *caz* inactivation in neurons and muscle cells. ***a-f,*** Immunostaining for caz and the neuronal nuclear marker elav on third instar larval ventral nerve cord revealed occasional caz inactivation in neurons when UAS-Cre is present in the caz^{lox} background (arrowheads), but not when UAS-FLP is present in the caz^{FRT} background. ***g-l,*** Immunostaining for caz on third instar larval body wall muscles along with DAPI staining of nuclei revealed occasional caz inactivation in muscles when UAS-Cre is present in the caz^{lox} background (arrowheads), but not when

UAS-FLP is present in the *caz*^{FRT} background.

SUPPLEMENTARY FIGURE S4:

Caz mutant pupal lethality is rescued by neuron-selective wild type caz expression. Note that the mere presence of UAS-caz also induces a slight but significant rescue of pupal lethality, probably attributable to leaky caz expression. Following genotypes are also shown in Figure 1e: WT, revertant, *caz*^{AttR}, *caz*^{2}, *caz*^{2}, elav-GAL4>UAS-caz, *caz*^{KO} and *caz*^{KO}, elav-GAL4>UAS-caz.

SUPPLEMENTARY FIGURE S5:

Full-length Western blot of which a cropped version is shown in Figure 1c. The blot was probed with primary antidodies against caz and β-tubulin (loading control).

SUPPLEMENTARY MOVIE LEGENDS

SUPPLEMENTARY MOVIE S1:

Pupal eclosion of wild type control flies. The movie is 10x sped up.

SUPPLEMENTARY MOVIE S2:

Pupal eclosion of *caz*^{2} flies. The movie is 10x sped up.

SUPPLEMENTARY MOVIE S3:

Motor deficits displayed by *caz*^{2} pharate adult flies that were dissected out of their pupal case when partially eclosed.

SUPPLEMENTARY MOVIE S4:

Pupal eclosion of an elav-GAL4>UAS-FLP fly.

SUPPLEMENTARY MOVIE S5:

Pupal eclosion of a *caz*^{FRT}, elav-GAL4>UAS-FLP fly. The movie is 8x sped up.

SUPPLEMENTARY MOVIE S6:

Motor performance of elav-GAL4>UAS-Cre (two left tubes), *caz*^{lox}; UAS-Cre (two middle tubes) and *caz*^{lox}, elav-GAL4>UAS-Cre (two right tubes) flies in a negative geotaxis climbing assay.

Supplementary Table S1: List of oligonucleotides used for cloning and PCR-genotyping.

Name	Sequence (5' to 3')
5'HR_FW	ATGCGGTACCGACAGTCCGATTGTCTTCTCCCACAACG
5'HR_REV	CTAGTCTAGACGTGTTAAAATTATACTTACGTTCCATG
3'HR_FW	TAGCGCTAGCCTTTGAATTCGGAAGTTGACATCATTCATTTCC
3'HR_REV	TCAGCCTCGAGGCCACGTTGTGGAGCATCTCATCGGTG
pABC_XbaI_FW	TTGTCTAGACAACATAACACCACCATACAGCAGCAGCAACATGTCG
pABC_XbaI_REV	TTATGTTGTCTAGACAAGCTTGTACGCGCCCGGGGAGCCCAAGG
XbaI_caz_FW	ATCATCTAGACGCATAAGTCGCAACGTGAATTGAAAACGATG
KpnI_caz_REV	GCACGGTACCCAGTGATTTTGATTTTCTCTATATATATATATATATAC AC
2xloxP FW	GACAAAGCTTATAACTTCGTATAATGTATGCTATACGAAGTTATTCTA GAGACAGGTACCATAACTTCGTATAATGTATGCTATACGAAGTTATC GTACGAAGG
2xloxP REV	CCTTCGTACGATAACTTCGTATAGCATACATTATACGAAGTTATGGT ACCTGTCTCTAGAATAACTTCGTATAGCATACATTATACGAAGTTATA AGCTTTGTC
2xFRT FW	GACAAAGCTTGAAGTTCCTATTCTCTAGAAAGTATAGGAACTTCCCA TGGGTACGGTACCGAAGTTCCTATTCTCTAGAAAGTATAGGAACTTC CGTACGAAGG
2xFRT REV	CCTTCGTACGGAAGTTCCTATACTTTCTAGAGAATAGGAACTTCGGT ACCGTACCCATGGGAAGTTCCTATACTTTCTAGAGAATAGGAACTTC AAGCTTTGTC
NcoI_caz_FW	ATCACCATGGCGCATAAGTCGCAACGTGAATTGAAAACGATG
Caz -1500 FW	TATTGCCATCTTGCACGTTT
caz intron3 REV	AGGGAAGGAAGGAGTTCGAC
caz RMCE FW	GCAGATGTAAGACTCCCAAGG
caz RMCE REV1	TCGCTGTAACTCAACAGCAG
caz RMCE REV2	ATCGCTACACCCTGCTAGAAAAAG

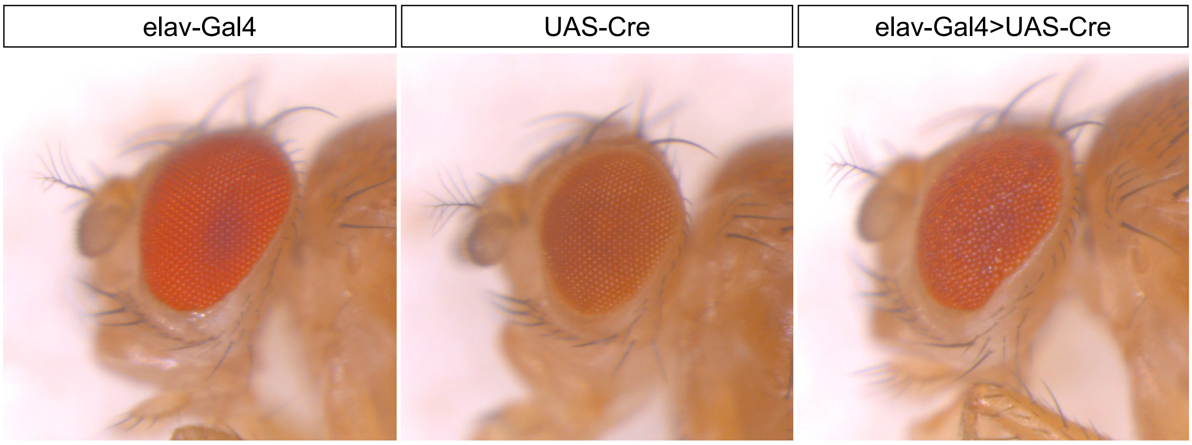

Figure S1

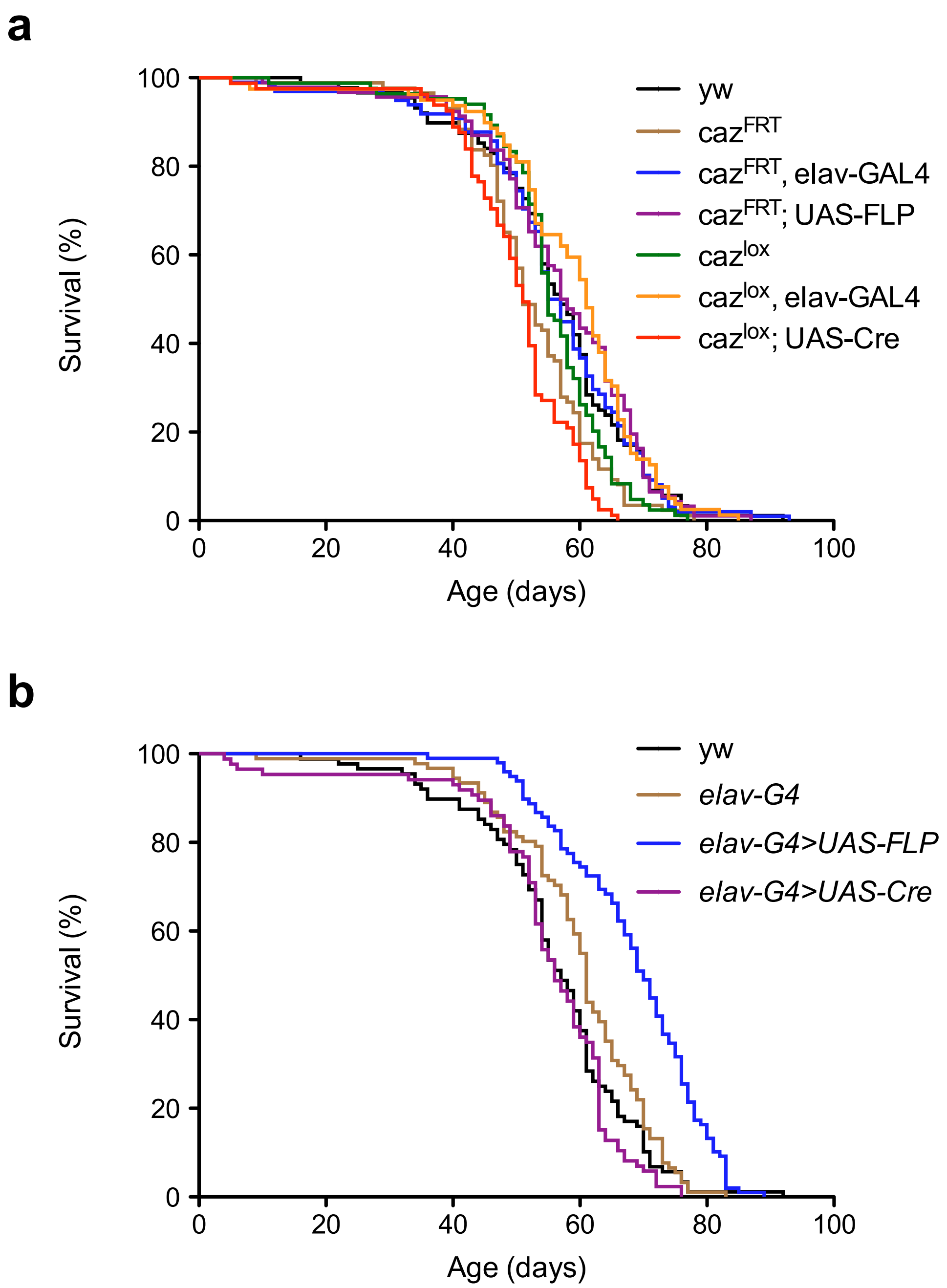
a
100
80
60
40
20
0
Survival (%)
0
20
40
60
80
100
Age (days)
yw
cazFRT
cazFRT, elav-GAL4
cazFRT; UAS-FLP
cazlox
cazlox, elav-GAL4
cazlox; UAS-Cre
b
100
80
60
40
20
0
Survival (%)
0
20
40
60
80
100
Age (days)
yw
elav-G4
elav-G4>UAS-FLP
elav-G4>UAS-Cre

Figure S2

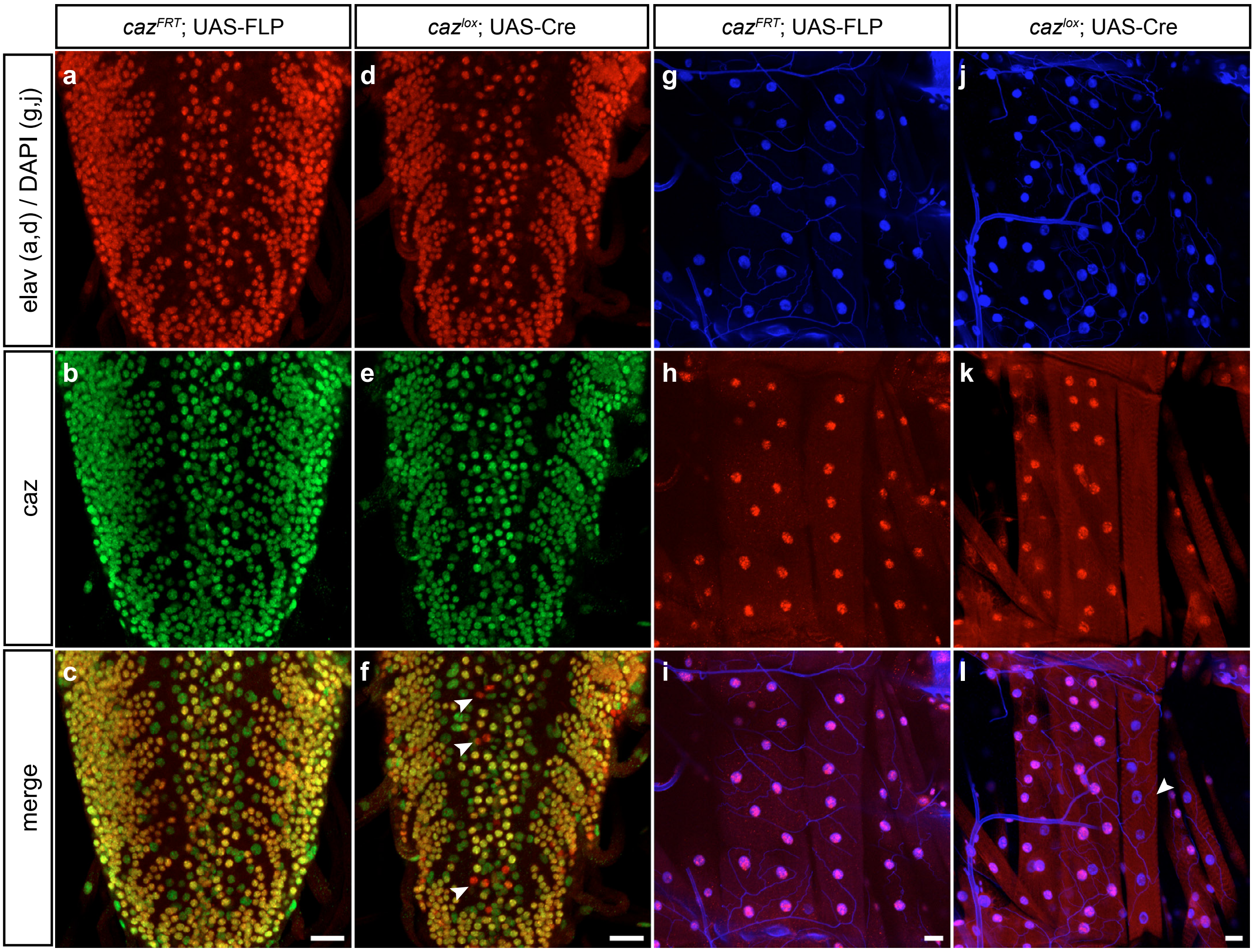

caz^FRT; UAS-FLP
caz^lox; UAS-Cre
caz^FRT; UAS-FLP
caz^lox; UAS-Cre
elav (a,d) / DAPI (g,j)
caz
merge
a
b
c
d
e
f
g
h
i
j
k
l

Figure S3

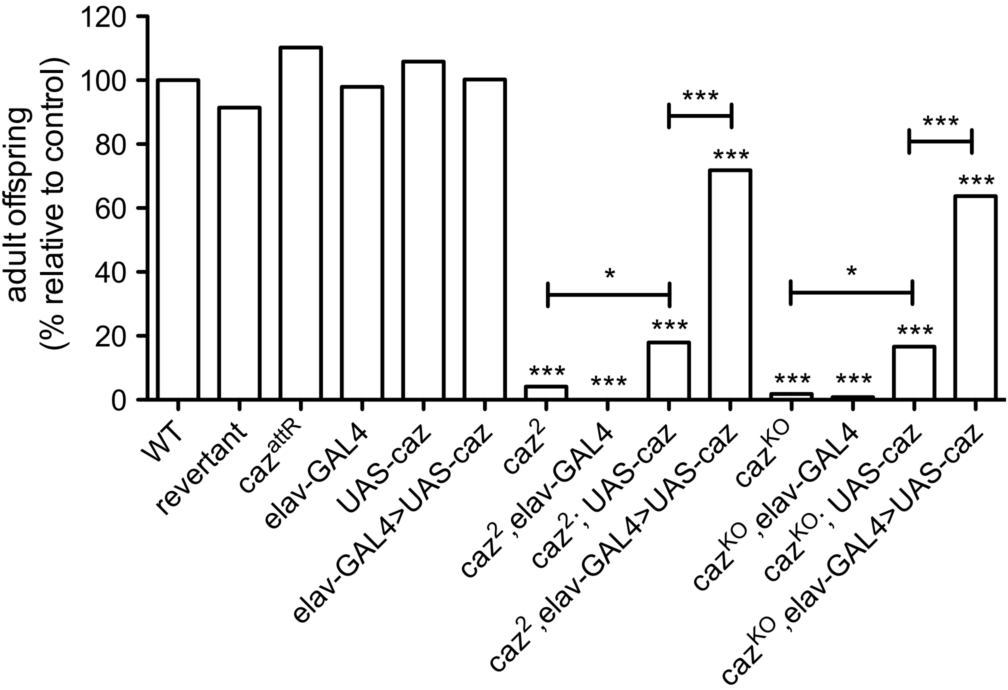
adult offspring
(% relative to control)
120
100
80
60
40
20
0
***
*
WT
revertant
caz^{attR}
elav-GAL4
UAS-caz
elav-GAL4>UAS-caz
caz^{2}
caz^{2},elav-GAL4
caz^{2}; UAS-caz
caz^{2},elav-GAL4>UAS-caz
caz^{KO}
caz^{KO},elav-GAL4
caz^{KO}; UAS-caz
caz^{KO},elav-GAL4>UAS-caz

Figure S4

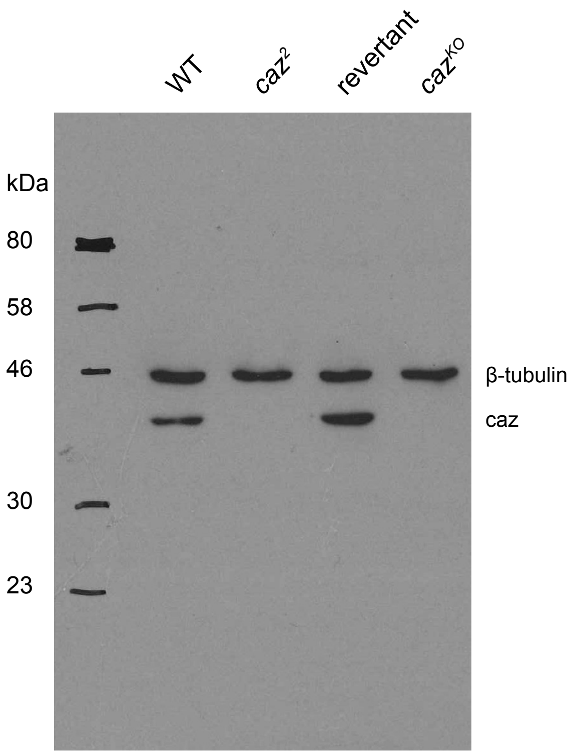

Figure S5
